# Tuberculin Treatment

**Published:** 1912-06

**Authors:** 


**Tuberculin Treatment.** By Clive Riviere, M.D., F.R.C.P., and Egbert Morland, M.D., B.Sc. Pp. xv, 277. London: Oxford Medical Publications. 1912.

The authors and the profession are alike to be congratulated on the publication of this book, which comes at a time when there is a well-marked, if belated, increase in the interest taken in the use of tuberculin in this country, and should do more than any work that has yet appeared to make clear the present position of tuberculin treatment and to act as a practical guide to the increasing number of those who wish to make use of it.

In striking contrast to so much that has been written on the subject, Drs. Riviere and Morland, though obviously enthusiasts, present their views in an extremely well-reasoned and well-balanced manner.

Whether the reader is or is not in accord with some of the theoretical parts of the book matters not, the point being that there is here placed within the reach of all who wish to use tuberculin a text-book, written in the simplest and most lucid manner, which will enable them with a little care and practice to select the right case and the right tuberculin and to regulate the dosage on fixed principles.

The book is divided into three portions, with a table of contents at the beginning of each. The first part deals with the general lines of tuberculin administration, and in it special attention is called to the essential unity of all tuberculins, and it is pointed out that this fact simplifies a good deal that has hitherto appeared obscure. At the same time, the two main varieties of tuberculins are indicated, namely those which contain endoplasm and those which are extracts only. The important clinical use which may be made of their difference in solubility is pointed out. The section on the relation between sensitiveness and tolerance, with diagrams, is particularly good. Under the heading "Standard of Dosage" the authors advocate the adoption of the cubic millimetre, and it is impossible to read their views without agreeing with them that "it is remarkable that it has not yet been widely adopted."

The table of equivalent doses under various systems of measurement is excellent. Very good and simple methods of preparing dilutions are given, the drop method being particularly ingenious. The problem of anaphylaxis is discussed, and attention called to the difference between it and increased tubercular sensitiveness.

One of the points on which the authors differ widely from most of the advocates of tuberculin is the question of the diagnostic value of one or more sub-cutaneous injections. They hold that a reaction, while indicating that infection with tuberculosis *has* occurred, does *not* indicate that there is tuberculous disease present requiring active treatment. They are also of opinion that repeated large doses may cause a reaction by inducing an artificial sensitiveness, while failure to react to smaller doses may be due to either deficient antibody formation or to the fact that tolerance has been developed.

Parts II and III deal respectively with "The Method of Immunisation with Tolerance (Koch)" and "The Method of Immunisation without Tolerance (Wright)," and herein lies the outstanding feature of the book, namely the clear distinction between the two large divisions of tuberculosis which can be treated with tuberculin. Part II then deals with the treatment of auto-toxic tuberculosis, in which class come practically all cases of pulmonary phthisis, and in which the essential factor is the production of tolerance. In Part III is discussed the treatment of localised tuberculosis, in which it is seldom necessary or advisable to attempt to induce tolerance by increasing doses given at short intervals. Failure to recognise these main divisions has led to much of the dissatisfaction felt with the results obtained in the past.

In Part II the sections on the principles of dosage are especially worthy of note. Three methods are discussed: (i) that of ignoring reactions, (ii) that of avoiding reactions, and (iii) that of utilising reactions. The authors advocate the last method, and agree with Turban, that if treatment is to be efficient it is impossible to avoid reactions altogether. They object to the pushing of tuberculin through the reaction limit, and give an excellent diagrammatic chart demonstrating their reasons.

When reactions do occur, "typhoid rest" on the lines laid down by Paterson must be strictly enforced, especially in febrile cases and those with secondary reactions.

A general reaction is not regarded as ever dangerous, but the "focal" may be. A "clinical" focal reaction should be avoided if possible. The local reaction may be used as a guide and often as a warning of the approach of a general reaction.

The uniform initial dose recommended in afebrile cases is ·001 c.m.m. of any tuberculin, original fluid. The section on increase of doses is very clear, and a chart (Chart xx), slightly altered from Lawrason Brown, gives a most ingenious and labour-saving scheme of dosage on various bases.

The questions of maximum dose and duration, of course, are clearly discussed, and in the next section—repetition of course—comes the other point, on which the authors differ widely from the generally accepted view among the experienced. They state that they "regard it as certain, alike in theory and practice, that reactivity may return without recrudescence of the disease and without any necessity for renewed treatment." The chart (Chart xxi) explaining the views held on this point is particularly interesting, and the black cross, indicating a fatal issue, must be making almost its first appearance in an English text-book.

The word "reactivity" is not satisfactory; it is almost impossible to dissociate it mentally from the idea of renewed activity of the disease. Perhaps "the power to react" or "reacting power" would be preferable.

The authors are of opinion that there are "no contra-indications of an absolute character," but somewhat modify this opinion later when discussing cases of severe mixed infection and those with much consolidation. Like other experts, they do not regard hæmoptysis as a contra-indication.

The combination of sanatorium treatment with tuberculin is highly recommended, and both writers have found the opsonic control of great use, but now only employ it as an aid in difficult problems. Then follow sections on the treatment of auto-toxic tuberculosis in children and on dispensary treatment, the advantages and disadvantages of the latter being made clear.

It is urged, as it is by all who have made extensive use of tuberculin, that patients lose their sputum, or the bacilli from their sputum, more rapidly and more frequently under specific treatment than under other methods, and that cases with the disease arrested are less liable to break down.

An interesting and instructive paragraph is devoted to the beneficial effects of tuberculin on symptoms in cases past hope of cure or even of arrest. Allusion is also made to the use of tuberculin as a febrifuge, given in very small doses in severe cases.

Want of space prevents a full notice of the third part of this book, which is devoted to the treatment of localised tuberculosis without the establishment of tolerance. It deals largely with disease in children, is written equally instructively and lucidly with the previous sections, and is equally worthy of close study; in fact, the subject-matter, dealing as it does largely with the measures which can be taken to render the hopeless case of phthisis an occasional rarity, is, if anything, more important than that of the rest of the book. The original diagram-charts, to one or two of which attention has been especially called, are uniformly excellent and easy of comprehension. The extensive bibliography will give some idea of the labour involved in the production of this little manual, which every practiser of physic should read and possess.

